# Piezoelectric cold atmospheric plasma inactivates dairy cattle bacteria and potentiates antibiotics in vitro

**DOI:** 10.1007/s00253-026-13843-9

**Published:** 2026-04-24

**Authors:** Jessica S. Ghodke, Dayane Lemos Teixeira, Daniel Enriquez-Hidalgo, Matt J. Bell, Olena Doran, Alexandros Ch. Stratakos

**Affiliations:** 1 Centre for Research in Sustainable Agri-Food and Environment, School of Applied Sciences, University of the West of England, Bristol, UK; 2 Animal and Agriculture Department, Hartpury University, Gloucester, UK; 3 Bristol Veterinary School, University of Bristol, Bristol, UK

**Keywords:** Piezoelectric cold plasma, Antimicrobial efficacy, Bacterial cell integrity, Antibiotic susceptibility, *E. coli*, *S. aureus*, *S. epidermidis*

## Abstract

**Abstract:**

Wound infection remains a major challenge in animal health and welfare, particularly in the farm environment, where skin injuries are prone to microbial contamination. However, reliance on antibiotics increases the risks of antimicrobial resistance and drug residues in animal products. Therefore, developing effective non-antibiotic-based approaches for wound decontamination is critically important and an emerging priority. In the current study, the antimicrobial efficacy of cold atmospheric plasma (CAP) was assessed against *Escherichia coli* P4, *Staphylococcus aureus* M60, and *Staphylococcus epidermidis* NCTC 11047 inoculated onto a bovine collagen-elastin matrix that approximates dermal tissue. Bacterial survivors (log CFU/cm^2^) were quantified after CAP exposure of 30–180 s at 1–2 cm source distances, alongside mechanistic assessments (viability staining, intracellular reactive oxygen species (ROS), extracellular lactate dehydrogenase (LDH), adenosine triphosphate (ATP) leakage, and lipid peroxidation). At 1 cm exposure distance, CAP reduced viable counts below detection limit after 120 s across all strains; at 2 cm, significant but distance-attenuated reductions were maintained. CAP exposure decreased viability in a treatment time-dependent manner, increased intracellular ROS, and compromised membrane integrity, consistent with envelope permeabilisation. A brief 30-s pre-exposure enhanced antibiotic susceptibility, approximately halving MICs and reducing MBCs by up to 75% for oxytetracycline (engemycin) and enrofloxacin. These findings indicate that piezoelectric CAP provides broad antimicrobial activity and potentiates antibiotic efficacy, supporting translational potential for on-farm wound hygiene and antibiotic stewardship.

**Key points:**

• *Piezoelectric CAP effectively reduces bacterial log CFU/cm*^*2*^*.*

• *Piezoelectric CAP compromises bacterial cell integrity and causes oxidative damage.*

• *CAP-pretreated bacteria showed enhanced antibiotic susceptibility.*

**Graphical abstract:**

**Figure:**
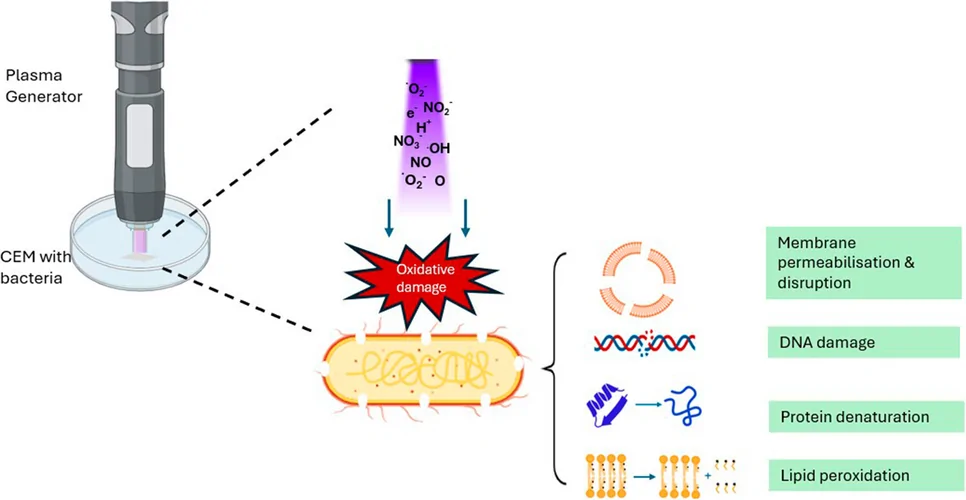

## Introduction

Skin injuries and wounds are a frequent occurrence in cattle, representing a significant concern for animal health and welfare. One of the primary causes arises from aggressive interactions within the herd, where horn-related trauma can result in deep lacerations and puncture lesions (Braun et al. 2016). Routine animal husbandry practices, such as dehorning and disbudding, represent significant sources of open wounds in cattle. In addition, individual identification with ear tags can result in localised cutaneous wounds. Furthermore, injuries can be incurred during transportation due to vehicle friction, poor handling practices, and high stocking densities (Alam et al. 2018). Similarly, animals can sustain dermal and epidermal punctures and abrasions while crossing or coming in contact with fences. Collectively, these factors are major causes of skin trauma, abrasions, bruises, and lacerations. Such lesions can be easily infected by microorganisms such as *Staphylococcus aureus*, *Streptococcus *spp., and coliforms like *Escherichia coli*, which are frequently associated with wound colonisation in livestock (Mala et al. 2021). If left untreated, these injuries can serve as an entry point for opportunistic pathogens, leading to secondary infections. Post-procedure wounds resulting from dehorning and disbudding are particularly prone to infection (Sheil et al. 2021), which can spread to adjacent tissues, becoming systemic, predisposing cattle to health complications such as acute sinusitis. Pathogens, including *E. coli*, *Arcanobacterium pyogenes*, *Pasteurella multocida*, *Trueperella pyogenes*, and anaerobes, have been identified as causative agents in such cases, significantly impairing recovery and wound healing (Divers 2009). Given the high incidence of such injuries and the associated risk of microbial infections, effective wound care is crucial for maintaining livestock welfare and the sustainability of agricultural operations. Although antibiotics are commonly used for alleviating most bacterial infections, concerns over antibiotic resistance and their residues in animal products highlight the need for alternative antimicrobial strategies (Mann et al. 2021). Increasing experimental evidence has proven CAP technology as an antimicrobial therapy and also a tool for tissue decontamination in medicinal applications (Daeschlein et al. 2015; Laroussi 2020; Bolgeo et al. 2023). CAP is a near-room-temperature ionised gas, consisting of reactive species, neutral particles, molecules, electrons, and other physical components, including electromagnetic field and weak thermal effect (Braný et al. 2020). Its antimicrobial activity is primarily attributed to reactive oxygen and nitrogen species (RONS), which impose physical and chemical stress on bacterial cells (Lv and Cheng 2022). These reactive species exert oxidative damage, targeting different cell components, causing DNA damage, protein denaturation, and lipid peroxidation. These alterations in cellular macromolecules culminate in the disruption of essential cellular functions, ultimately leading to bacterial cell death (Das et al. 2022).

This study differs from prior CAP work that predominantly employs dielectric-barrier discharge or gas-fed plasma jets. A piezoelectric, air-fed, handheld generator used in this study requires low-voltage power, eliminating the need for an external gas supply and enabling true portability. The primary aim of the study was to evaluate CAP as a potential antimicrobial treatment for wound decontamination. Although direct application of CAP to cattle wounds was not performed in the current study, bacteria isolated from dairy cattle that could potentially infect wounds were used as representative strains. Therefore, the objectives of the study were (i) to assess CAP’s antimicrobial efficacy against bacteria isolated from dairy cattle using a collagen-elastin matrix that simulates skin; (ii) to elucidate the underlying mechanism of CAP’s antimicrobial activity with a focus on its effect on bacterial cell integrity; and (iii) to assess the antimicrobial effect of CAP in combination with antibiotics against these bacterial strains.

## Materials and methods

### CAP generation and operating parameters

CAP was generated using a piezoelectric direct discharge generator (PiezoBrush® PZ3; Relyon Plasma GmbH, Germany) equipped with a standard module. The plasma generator operated using a 24-V AC-DC adapter and a CeraPlas F piezoelectric transformer at a frequency of 50 kHz. The generator utilised atmospheric ambient air as the gas source (8 L/min), with no external gas feed supplied. An in-built fan propelled plasma toward the outlet, enabling uniform delivery onto the matrix surface. The treatment duration ranged from 30 to 180 s as detailed below. A detailed schematic representation of the plasma generator is presented by Korzec et al. (2021).

### Bacterial culture preparation

*E. coli* P4, *S. aureus* M60 (isolated from dairy cattle), and *S. epidermidis* NCTC 11047 were used in this study. These strains were selected to represent clinically relevant bovine wound-associated bacteria, including both Gram-negative (*E. coli* P4) and Gram-positive (*S. aureus* M60, *S. epidermidis* NCTC 11047). These strains were used from frozen stock stored at −80 °C in Cryoinstant vials with porous beads (Microbank, Pro-Lab Diagnostics, UK). A single bead was aseptically transferred into sterile Brain Heart Infusion (BHI) broth (Oxoid, UK) and incubated at 37 °C for 24 h to revive the culture. Subsequently, 100 μL of culture was inoculated into 10 mL sterile BHI and incubated at 37 °C for 24 h. To prepare the working culture, inoculated BHI broth for each bacterial strain was centrifuged at 6500 × g for 10 min at 20 °C, washed twice with phosphate-buffered saline (PBS, pH 7.4; Oxoid, UK), and resuspended in 10 mL maximum recovery diluent (MRD, Oxoid, UK) (~ 9 log CFU/mL). The suspension was further serially diluted in MRD to obtain the desired bacterial concentration (6 log CFU/mL). Bacterial concentrations were confirmed by viable plate count. No McFarland standard was used; instead, bacterial suspensions were standardised based on colony forming unit (CFU/mL) enumeration. This preparation method was used consistently across all experiments.

### Evaluation of CAP antimicrobial activity

The antimicrobial activity of CAP was assessed by exposing the test bacteria to CAP. Two key operational parameters, exposure time and distance (between the plasma source and bacteria), were investigated to determine the optimal conditions for maximum bacterial inactivation. The antimicrobial assay was conducted following a modified version of the protocol described by Boekema et al. (2021). To simulate animal skin and evaluate CAP’s antimicrobial activity in a more realistic setting (physiologically relevant), a collagen-elastin matrix (CEM) (Matriderm® Germany) with a thickness of 1 mm was utilised. CEM is a single-layer dermal matrix composed of bovine collagen and elastin, commonly used in wound treatment. This approach allowed us to assess the effectiveness of CAP in conditions more closely resembling actual skin surfaces. *E. coli* P4, *S. aureus* M60, and *S. epidermidis* NCTC11047 were grown in BHI at 37 °C for 24 h and bacterial dilutions were prepared as mentioned in the Bacterial culture preparation section. Under aseptic conditions, the CEM membrane was cut into small pieces of 1 × 1 cm^2^. Each CEM piece was a single bacterial strain (either *E. coli* P4, *S. aureus* M60, or *S. epidermidis* NCTC 11047) and inoculated with 10 µL of bacterial suspension containing approximately 6 log CFU/mL. Experiments were conducted separately for each bacterial strain, and no mixed bacterial culture was used. The inoculated CEMs were then exposed to CAP for 30–180 s at intervals of 30 s, with plasma-CEM distances maintained at 1 and 2 cm. Following CAP treatment, each CEM piece was suspended in maximum recovery diluent (MRD; Oxoid™, UK) and vortexed to release the attached bacterial cells. The resulting cell suspension was then serially diluted, and 100 µl from 10^−1^ to 10^−3^ dilutions was plated on sterile BHI agar and incubated at 37 °C for 24 h. In addition, 10^0^ µl of the undiluted sample (100 dilution) was plated to determine the limit of detection. The control samples were processed identically, except with no CAP treatment. The viable bacterial count was expressed as log CFU/cm^2^ and was calculated after 24 h for both treated and control samples. The reduction in the log CFU/cm^2^ was the difference between the log CFU/cm^2^ of the control and treated bacterial samples. The formula for the lowest detection limit is log_10_ (No. of bacterial colonies/volume plated × dilution factor). Based on the preliminary optimisation experiments (see Results section), a distance of 1 cm between CAP and bacteria was selected for all the subsequent experiments.

### Bacterial viability assay

Bacterial cell viability following CAP treatment was evaluated using the LIVE/DEAD™ BacLight™ bacterial viability kit, Invitrogen (Cat# L13151, UK). A 24-h culture of *E. coli* P4, *S. aureus* M60, and *S. epidermidis* NCTC11047 was centrifuged at 6500 × g for 10 min at 20 °C, and the resulting pellets were resuspended in 10 mL of sterile saline. CAP treatment was applied to each bacterial suspension as described in the Evaluation of CAP antimicrobial activity section. The dye solutions SYTO 9 (6 µM) and propidium iodide (30 µM) were prepared according to the manufacturer’s instructions. The treated bacterial suspension sample and dye solution (consisting of SYTO 9 and propidium iodide, PI) were mixed in a 1:1 ratio and incubated at room temperature in the dark for 20 min. The assay was performed in a 96-well flat-bottom cell culture microplate (Corning™ Costar™, USA), and the fluorescence was measured using a microplate reader (VANTAstar; BMG LABTECH, Germany). SYTO 9 fluorescence (excitation 485 nm and emission 530 nm) was used to quantify viable cells, while PI fluorescence (excitation 485 nm and emission 630 nm) was used to quantify membrane-compromised or dead cells. Control samples were processed identically without CAP treatment (Yadav and Roopesh 2023). The percentage of surviving bacteria was calculated using the following formula.

$${\% live cells}=\left(100\times \frac{\text{SYTO }9}{\mathrm{PI}}\right)\div \left(1+\frac{\text{SYTO }9}{\mathrm{PI}}\right)$$

where SYTO 9 represents the fluorescence at 485/530 (live bacteria), and PI represents the fluorescence at 485/630 nm (dead bacteria) (Stiefel et al. 2015).

### Quantification of CAP-induced intracellular reactive oxygen species (ROS)

Intracellular ROS levels in *E. coli* P4, *S. aureus* M60, and *S. epidermidis* NCTC11047 were detected using 6-carboxy-2′,7′-dichlorodihydrofluorescein diacetate (H_2_DCFDA; Merck, UK)*.* H_2_DCFDA is a non-fluorescent cell-permeable dye that is hydrolysed by intracellular esterases and subsequently oxidised by ROS to produce the green, fluorescent 2′,7′-dichlorofluorescein (DCF)*.* For CAP treatment of the bacterial suspension, 500 µL of bacterial suspension (approximately 6 log CFU/mL) was added to a well of a 6-well plate (Corning™ Costar™, USA). The bacterial suspension formed a thin layer covering the base of the well to ensure effective plasma exposure. The distance between the plasma source and the liquid surface was maintained at 1 cm.

A 100 µL aliquot of CAP-treated bacterial suspension was mixed with 50 µL of 20 µM H_2_DCFDA and incubated in the dark for 20 min. CAP-untreated bacterial suspension was used as the control. The assay was performed in a 96-well flat-bottom cell culture microplate (Corning™ Costar™, USA), and the fluorescence was measured using a microplate reader (VANTAstar; BMG LABTECH, Germany) with excitation and emission wavelengths at 480 nm and 530 nm, respectively (Yang et al. 2021b; Lv and Cheng 2022).

### Measurement of CAP-induced lipid peroxidation

The bacterial suspensions of each strain were treated with CAP under the same conditions described in the Quantification of CAP-induced intracellular reactive oxygen species (ROS) section (500 µL suspension, 1 cm distance). Lipid peroxidation was assessed by quantifying malondialdehyde (MDA), a by-product of oxidative degradation of lipids. MDA content of 100 µL CAP-treated bacterial suspensions of *E*. *coli*, *S. aureus*, and *S. epidermidis* was measured using Lipid Peroxidation (MDA) Assay Kit (Sigma-Aldrich, UK) as per the manufacturer’s instructions. CAP-treated bacterial suspensions were mixed with thiobarbituric acid (TBA) and incubated at 95 °C for 60 min, followed by cooling in an ice bath for 10 min. Absorbance was measured at 532 nm using a microplate reader (VANTAstar; BMG LABTECH, Germany). MDA concentrations were determined using a standard MDA calibration curve.

### Evaluation of bacterial membrane integrity

The bacterial suspensions of each strain were treated with CAP under the same conditions described in the Quantification of CAP-induced intracellular reactive oxygen species (ROS) section (500 µL suspension, 1 cm distance). The effect of CAP on bacterial cell integrity was assessed by measuring extracellular LDH activity and extracellular ATP content. LDH activity in CAP-treated and control samples was quantified using Invitrogen CyQUANT LDH Cytotoxicity Assay kit (Thermo Fisher Scientific, UK) as per the manufacturer’s instructions*.* Briefly, the reaction mixture was added to the 100 µL of CAP-treated bacterial suspensions, incubated for 30 min, and then the stop solution was added before measuring the absorbance at 480 and 680 nm using a microplate reader (VANTAstar; BMG LABTECH). The final absorbance values were obtained by subtracting readings at 680 nm (background) from those at 480 nm (Maybin et al. 2023). Extracellular ATP content from 100 µL CAP-treated bacterial suspension of *E. coli* P4, *S. aureus* M60, and *S. epidermidis* NCTC11047 was quantified using a firefly luciferase-based bioluminescent ATP assay kit (Invitrogen™, UK) in accordance with the manufacturer’s instructions. To ensure measurement of only extracellular ATP, the bacterial cell lysis step was omitted. A standard curve was generated by recording luminescence from a series of ATP standards using a microplate reader (VANTAstar; BMG LABTECH). Luminescence from CAP-treated and control bacterial suspensions was then measured under identical conditions, and the ATP concentrations were calculated from the standard curve (Maybin et al. 2023).

### Antibiotic susceptibility following CAP pretreatment

The effect of CAP pretreatment on bacterial susceptibility to the antibiotics engemycin and enrofloxacin was investigated in *E. coli* P4, *S. aureus* M60, and *S. epidermidis* NCTC11047 by determining the minimum inhibitory concentration (MIC) and minimum bactericidal concentration (MBC). The bacterial suspensions of (500 µL suspension, ~6 log CFU/mL) *E*. *coli*, *S. aureus*, and *S. epidermidis* were exposed to CAP for 30 s before antibiotic susceptibility testing. CAP treatment of 30 s showed no significant reduction in bacterial log CFU/cm^2^ and therefore was considered an ideal duration for CAP pretreatment. Following CAP treatment, bacterial suspensions were immediately subjected to MIC and MBC determination. The MIC values for control and CAP-pretreated bacteria were determined by the broth microdilution method according to the protocol described by Balouiri et al. (2016). Antibiotic (engemycin and enrofloxacin) concentrations were used in the range of 0.125 to 512 µg/mL. MBC was determined by plating 100 µL from wells showing no visible growth onto sterile BHI agar, followed by incubation at 37 °C for 24 h. BHI broth inoculated with bacteria served as a positive control, while uninoculated BHI served as a negative control for both CAP-treated and untreated samples (Maybin et al. 2023).

### Statistical analysis

Each experiment was performed in biological triplicate, with three technical repeats per biological replicate (*n* = 9). The results are represented as mean values ± standard deviation (SD). The data generated were statistically analysed using Minitab (version 22.3, Minitab software, USA). For individual comparisons within each experimental method (for example, the effect of CAP treatment duration within a single bacterial strain), one-way analysis of variance (ANOVA) followed by Tukey’s post hoc test was applied at a significance level of *p* < 0.05. To evaluate the combined effects of CAP treatment duration and bacterial strains, a two-way ANOVA was performed, followed by a post hoc test (at a significance level of *p* < 0.05), including interaction effects between variables. MIC and MBC values were determined using standard broth microdilution assays and are reported as discrete values based on serial double dilution steps. As identical values were obtained across all replicates, no statistical analysis was performed for MIC/MBC data.

## Results

### Antimicrobial efficacy of CAP on a collagen-elastin matrix

**Fig. 1:**
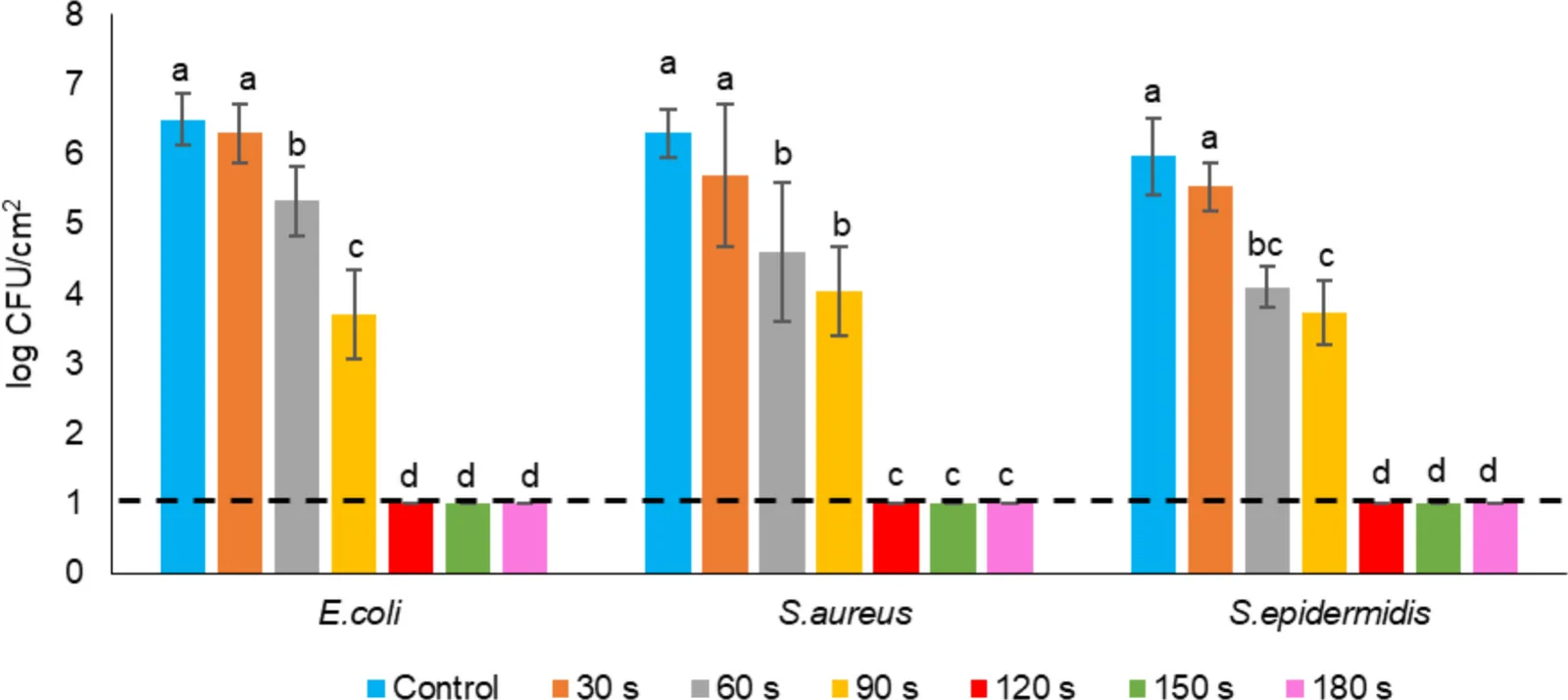
Effect of CAP treatment on bacteria (log CFU/cm^2^) in *E. coli* P4, *S. aureus* M60, and *S. epidermidis* NCTC11047. CAP was applied for a duration of 30–180 s at 1 cm. CAP-untreated samples served as the controls. The bars in the graph represent the mean value of log CFU/cm^2^ with standard deviation indicated by error bars, derived from three independent experiments, each conducted in triplicate (*n* = 9). The dotted line indicates the bacterial detection limit (1 log CFU/cm^2^). Bars with different superscript letters differ significantly (*p* < 0.05) within each bacterial group

The antimicrobial activity of CAP was first quantified by assessing the reduction in viable counts of Gram-negative and Gram-positive bacterial populations under varying treatment conditions. Figure 1 presents the effect of CAP exposure time on *E. coli* P4, *S. aureus* M60, and *S. epidermidis* NCTC11047 when the distance from the CAP source was 1 cm. As shown in Fig. 1, CAP exposure for 30 s did not produce a significant effect on any of the bacterial strains tested. However, increasing the exposure time to 60 s resulted in a significant reduction in CFU/cm^2^ of 1.2 ± 0.5 in *E. coli*, 1.7 ± 1.0 in *S. aureus*, and 1.8 ± 0.3 in *S. epidermidis*, compared to the corresponding controls (*p* < 0.05). A further reduction in bacterial log CFU/cm^2^ was observed after 90-s treatment, particularly in *E. coli* with a decrease of 2.8 ± 0.6, followed by 2.3 ± 0.6 in *S. aureus*, and 2.2 ± 0.4 log CFU/cm^2^ in *S. epidermidis* (*p* < 0.05) as compared to controls. An increase in the CAP treatment time to 120 s or longer reduced the bacterial log CFU/cm^2^ below the detection limit of 1 log CFU/cm^2^ (*p* < 0.05) in all bacterial strains tested.

**Fig. 2:**
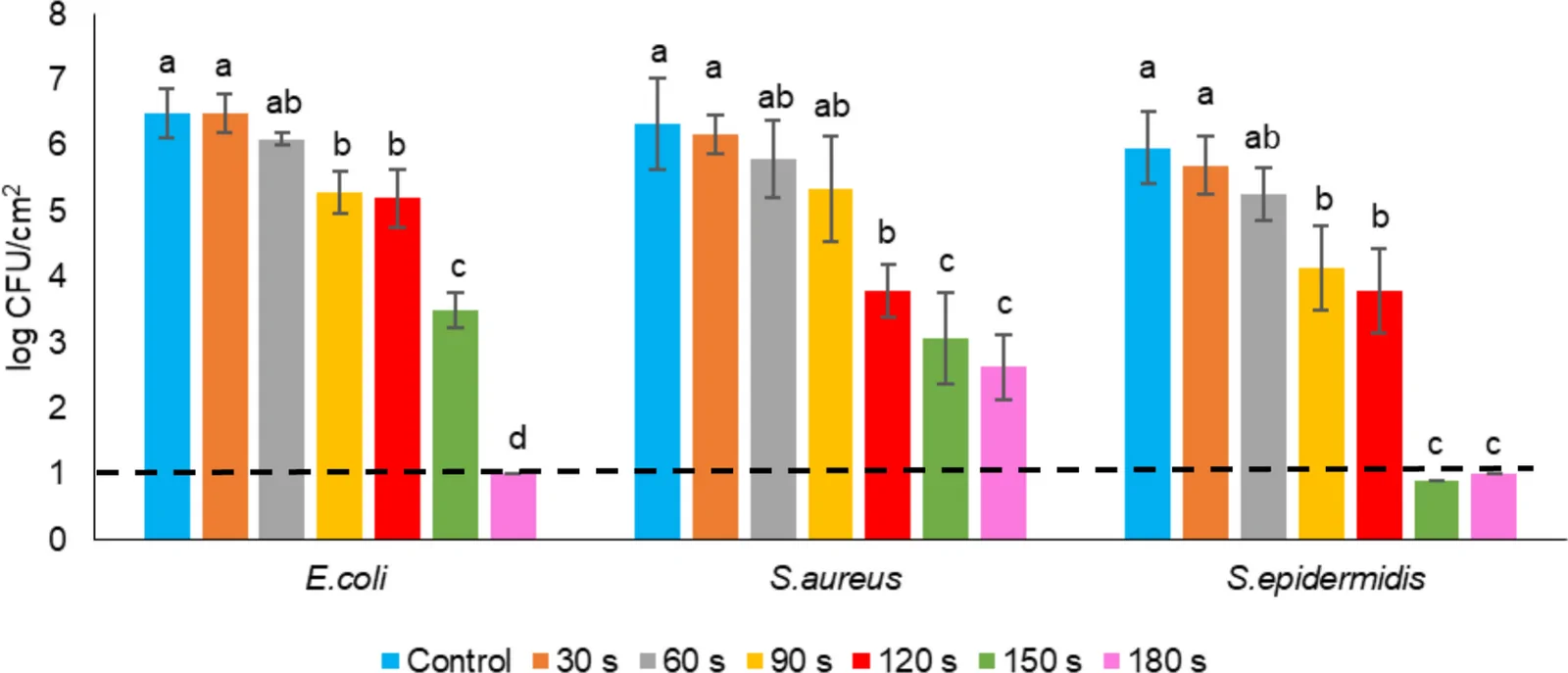
Effect of CAP treatment on bacteria (log CFU/cm^2^) in *E. coli* P4, *S. aureus* M60, and *S. epidermidis* NCTC11047. CAP was applied for a duration of 30–180 s at 2 cm. CAP-untreated samples served as the controls. The bars in the graph represent the mean value of log CFU/cm^2^ with standard deviation indicated by error bars, derived from three independent experiments, each conducted in triplicate (*n* = 9). The dotted line indicates the bacterial detection limit (1 log CFU/cm^2^). Bars with different superscript letters differ significantly (*p* < 0.05) within each bacterial group

When the distance between the CAP source and bacteria was increased to 2 cm, in this case as well, all bacterial strains demonstrated a time-dependent reduction in log CFU/cm^2^; however, the reduction levels were smaller as compared to 1 cm (Fig. 2). CAP exposure for 30 s showed reductions in log CFU/cm^2^; however, these were not statistically significant (*p* > 0.05). Extending the treatment to 60 s further reduced viable counts; however, the difference remained nonsignificant relative to the untreated controls. By 90 s, reductions were evident, with significant (*p* < 0.05) decreases of 1.2 ± 0.3, 1.0 ± 0.8, and 1.8 ± 0.6 log CFU/cm2 in *E. coli*, *S. aureus*, and *S*. *epidermidis*, respectively, compared to control. Increasing the treatment time to 120 s further enhanced the antimicrobial effect, yielding a reduction in cell count, with the highest reduction of 2.5 ± 0.4 log CFU/cm^2^ in *S. aureus*, followed by 2.1 ± 0.6 log CFU/cm^2^ in *S. epidermidis*, and finally 1.3 ± 0.4 log CFU/cm^2^ in *E. coli*. With CAP exposure extended to 150 s, a significant reduction in bacterial counts was observed across all strains (*p* < 0.05). Notably, *S. epidermidis* exhibited the highest reduction, with the CFU/cm^2^ falling below the detection limit (1 log CFU/cm^2^), followed by a reduction of 3.3 ± 0.7 log CFU/cm^2^ in *S. aureus* and 3.0 ± 0.2 log CFU/cm^2^ in *E. coli.* Finally, when the CAP treatment duration was increased to 180 s, the cell counts of *E. coli* were below the detection limit; however, *S. aureus* maintained a viable cell population of 2.6 ± 0.5 log CFU/cm^2^ with a nonsignificant reduction (*p* > 0.05) compared to 150-s treatment. Overall, both CAP treatment distances exhibited the same time-dependent bacterial inactivation; however, the reduction in bacterial log CFU/cm^2^ was lower when the CAP source was positioned at 2 cm. Based on these findings, a distance of 1 cm was selected for all subsequent experiments.

### Impact of CAP on bacterial viability

**Fig. 3:**
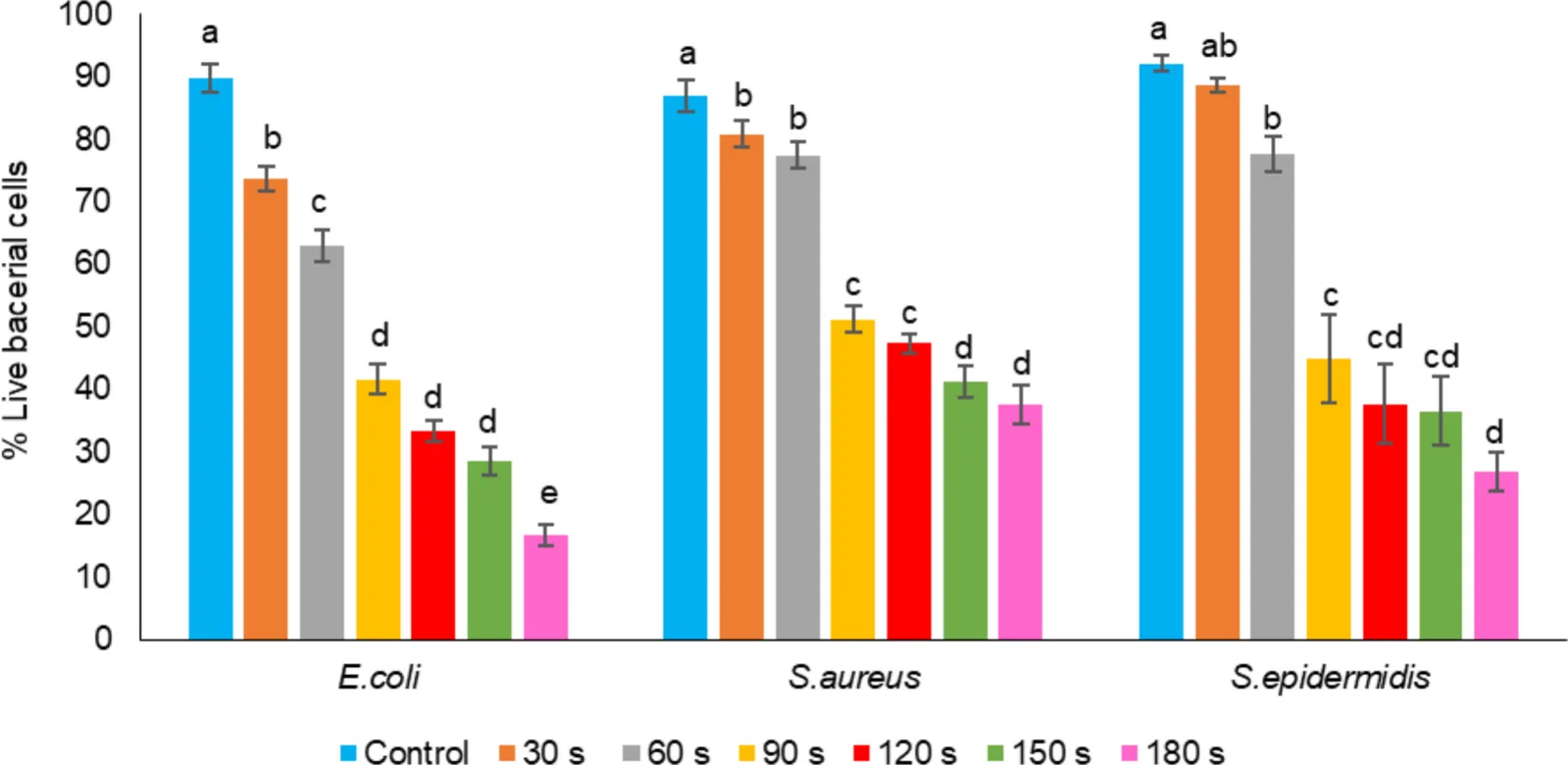
Effect of CAP treatment on bacterial viability (%) in *E*. *coli* P4, *S. aureus* M60, and *S. epidermidis* NCTC11047. CAP was applied for a duration of 30–180 s at 1 cm. CAP-untreated samples served as the controls. The bars in the graph represent the mean value of % live bacterial cells with standard deviation indicated by error bars, derived from three independent experiments, each conducted in triplicate (*n* = 9). Bars with different superscript letters differ significantly (*p* < 0.05) within each bacterial group

Given the reduction in viable bacterial counts following CAP exposure, further investigation was conducted to evaluate its impact on overall bacterial viability. Figure 3 presents the effect of CAP treatment on bacterial viability assessed using PI/SYTO fluorescence staining. As the CAP treatment time increased, a significant reduction (*p* < 0.05) in bacterial viability was observed in all bacterial strains compared to the controls. At 30 s, CAP treatment significantly reduced the viability of *E. coli* and *S. aureus* by 17.9% ± 2.2 and 6.9% ± 2.5, respectively; however, the reduction in *S. epidermidis* was not statistically significant compared to the control. After 60 s of CAP treatment, the viability of *E*. *coli* decreased by 29.8% ± 2.5, which was significantly greater than the reductions observed in *S. aureus* (10.8% ± 2.2) and *S. epidermidi*s (15.8% ± 2.7). A 90-s CAP treatment resulted in a significant decrease in bacterial cell viability (*p* < 0.05), with a 35.6% ± 2 decrease in *S. aureus* and ~50% decrease in *E. coli* and *S. epidermidis*. A further decline in viability (*p* < 0.05) was observed at 120-s CAP treatment, with a decrease of 56.3% ± 1.6 in *E. coli*, followed by 54.4% ± 6.3 in *S. epidermidis*, with the least reduction observed in *S. aureus* by 39.6% ± 1.5. Increasing the treatment time to 150 s did not show any statistically significant reduction in *E. coli* and *S. epidermidis* compared with the 120-s treatment; however, *S. aureus* exhibited a reduction (*p* < 0.05) of 45.7% ± 2.5. The most significant reduction occurred after 180 s, with *E. coli* viability reduced by 73.0% ± 1.7, followed by 65.9% ± 3.0 in *S. epidermidis* and 49.4% ± 3.0 in *S. aureus* as compared to the controls.

### Intracellular ROS level in CAP-treated bacteria

**Fig. 4:**
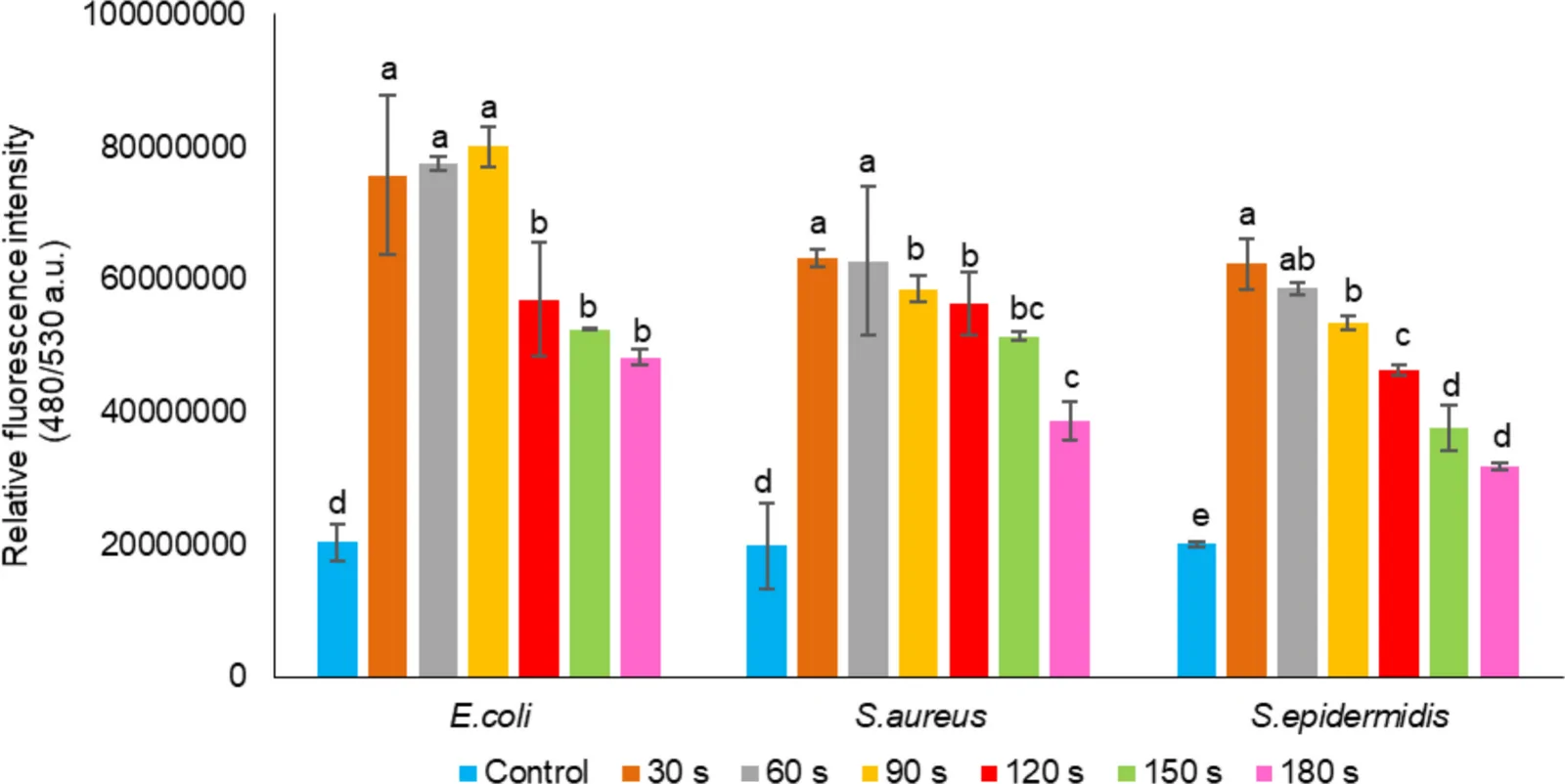
Effect of cold atmospheric plasma (CAP) treatment on intracellular ROS levels in *E*. *coli* P4, *S. aureus* M60, and *S. epidermidis* NCTC11047. CAP was applied for a duration of 30–180 s at a distance of 1 cm. CAP-untreated samples served as the controls. The bars in the graph represent the mean value of fluorescence intensity with standard deviation indicated by error bars, derived from three independent experiments, each conducted in triplicate (*n* = 9). Bars with different superscript letters differ significantly (*p* < 0.05) within each bacterial group

As CAP treatment resulted in a marked reduction in bacterial viability, consistent with the findings in the Impact of CAP on bacterial viability section, subsequent evaluation was conducted to elucidate whether oxidative stress contributed to the observed antimicrobial effect. To assess CAP-induced oxidative stress, the intracellular level of ROS in bacteria was evaluated. The ROS levels were significantly higher (*p* < 0.05) in bacterial strains post CAP treatment (30–180 s) as compared to untreated controls (Fig. 4). However, a significant elevation in intracellular ROS level was observed between 30 and 90 s in *E. coli*, with peak levels detected at 90 s as compared to the control. The concentration of ROS dropped (*p* < 0.05) after 90 s, and further CAP exposure beyond this point led to a progressive decline in intracellular ROS intensity, with the lowest at 180 s. A comparable trend in intracellular ROS accumulation was observed in both *S. aureus* and *S*. *epidermidis* following CAP treatment. In *S. aureus*, a spike in intracellular ROS occurred between 30 and 60 s, with levels significantly higher (*p* < 0.05) than the control, before decreasing over the remaining treatment duration. Similarly, *S. epidermidis* exhibited an increase in intracellular ROS immediately after 30 s, followed by a steady decrease as CAP treatment prolonged. Notably, the intracellular ROS content of *E. coli* was significantly (*p* < 0.05) higher than that of *S. aureus* and *S. epidermidis*.

### Lipid peroxidation in CAP-treated bacteria

**Fig. 5:**
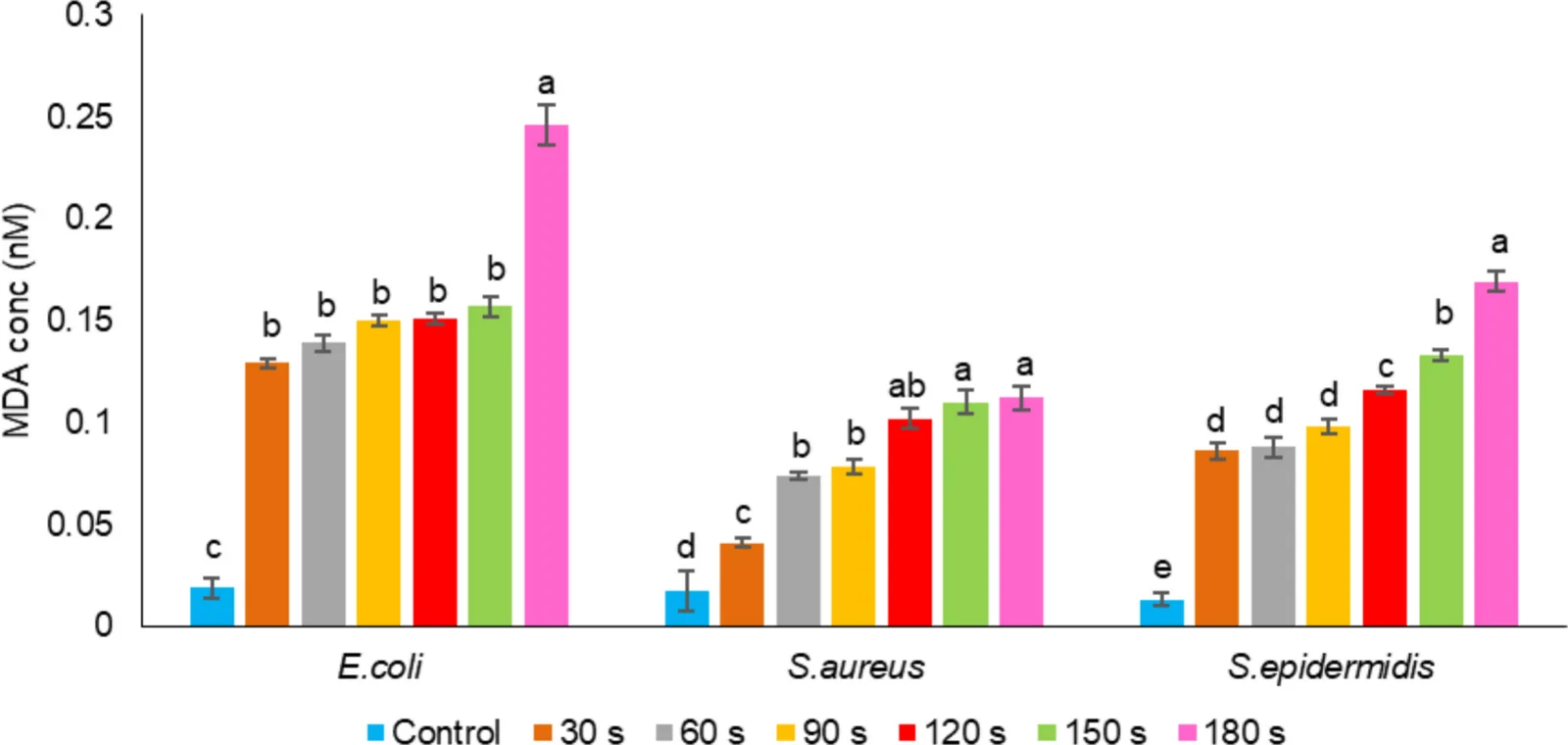
MDA in cell culture supernatant post CAP treatment in *E*. *coli* P4, *S. aureus* M60, and *S. epidermidis* NCTC11047. CAP was applied for a duration of 30–180 s at a distance of 1 cm. CAP-untreated samples served as the controls. The bars represent the mean concentration of MDA with standard deviation indicated by error bars derived from three independent experiments, each conducted in triplicate (*n* = 9). Bars with different superscript letters differ significantly (*p* < 0.05) within each bacterial group

After observing a pattern of elevated ROS followed by a decline, further investigation was focused on assessing whether the reactive species initiated oxidative damage to the membrane lipids. Lipid peroxidation is a key indicator of oxidative damage to the bacterial membrane. Lipid oxidation induced by CAP was assessed by measuring MDA in *E. coli*, *S. aureus*, and *S. epidermidis*. Controls exhibited low levels of MDA and were considered the baseline. CAP exposure resulted in a significant (*p* < 0.05), time-dependent increase in MDA concentration in all bacterial strains, indicating enhanced lipid peroxidation following CAP treatment (Fig. 5). In *E. coli*, MDA levels increased significantly after 30 s of CAP exposure compared to the control and continued to rise progressively with increasing treatment duration, reaching the highest level after 180 s. A similar trend was observed in *S. aureus*, where CAP treatment induced a gradual increase in MDA concentration, with statistically significant elevations observed from 30 s onwards and peak levels recorded after 150–180-s exposure. In *S. epidermidis*, CAP treatment also resulted in a steady, time-dependent increase in MDA levels, with significant lipid peroxidation evident from 30 s and the highest MDA accumulation observed following 180-s exposure.

### Effect of CAP on bacterial cell integrity

#### LDH release following CAP treatment

**Fig. 6:**
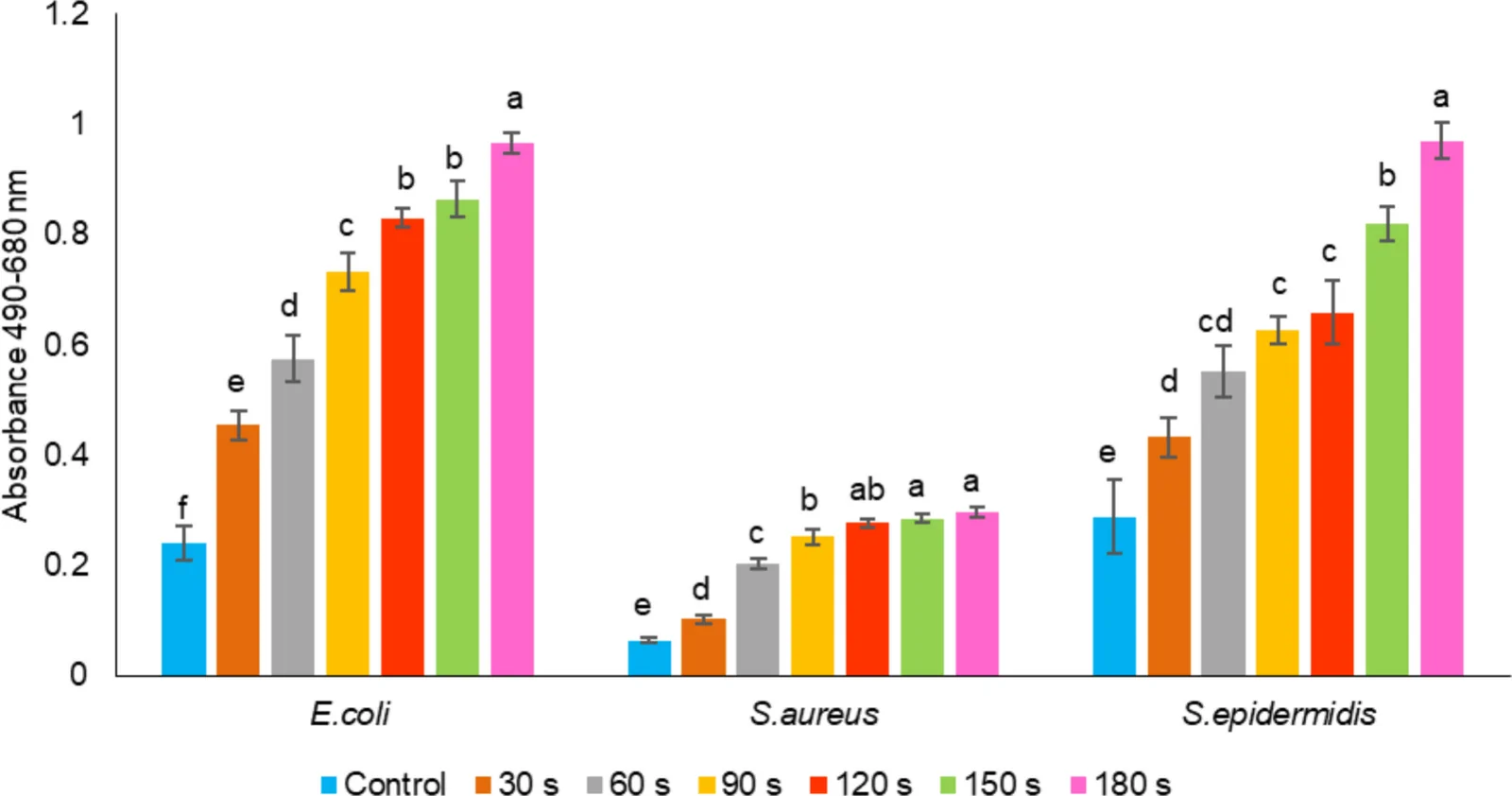
LDH activity post CAP treatment in *E*. *coli* P4, *S. aureus* M60, and *S. epidermidis* NCTC11047. CAP was applied for a duration of 30–180 s at a distance of 1 cm. CAP-untreated samples served as the controls. The bars represent the mean value of absorbance (490–680 nm) with standard deviation indicated by error bars, derived from three independent experiments, each conducted in triplicate (*n* = 9). Bars with different superscript letters differ significantly (*p* < 0.05) within each bacterial group

To determine whether lipid peroxidation resulted in compromised membrane permeability, the amount of LDH released was quantified as an indicator of cytoplasmic leakage. Extracellular LDH activity was measured to evaluate the response of *E. coli*, *S. aureus*, and *S. epidermidis* to CAP exposure. In the control groups, LDH levels were minimal across all strains. Following CAP treatment, LDH activity increased progressively with the treatment duration in all bacterial strains and was significantly higher (*p* < 0.05) than the controls at all time points (Fig. 6). The highest LDH activity was observed following 180-s CAP treatment in all bacterial strains. It is noteworthy that the LDH activity in *S. aureus* was significantly lower than that of *E. coli* and *S. epidermidis*.

#### ATP release following CAP treatment

**Fig. 7:**
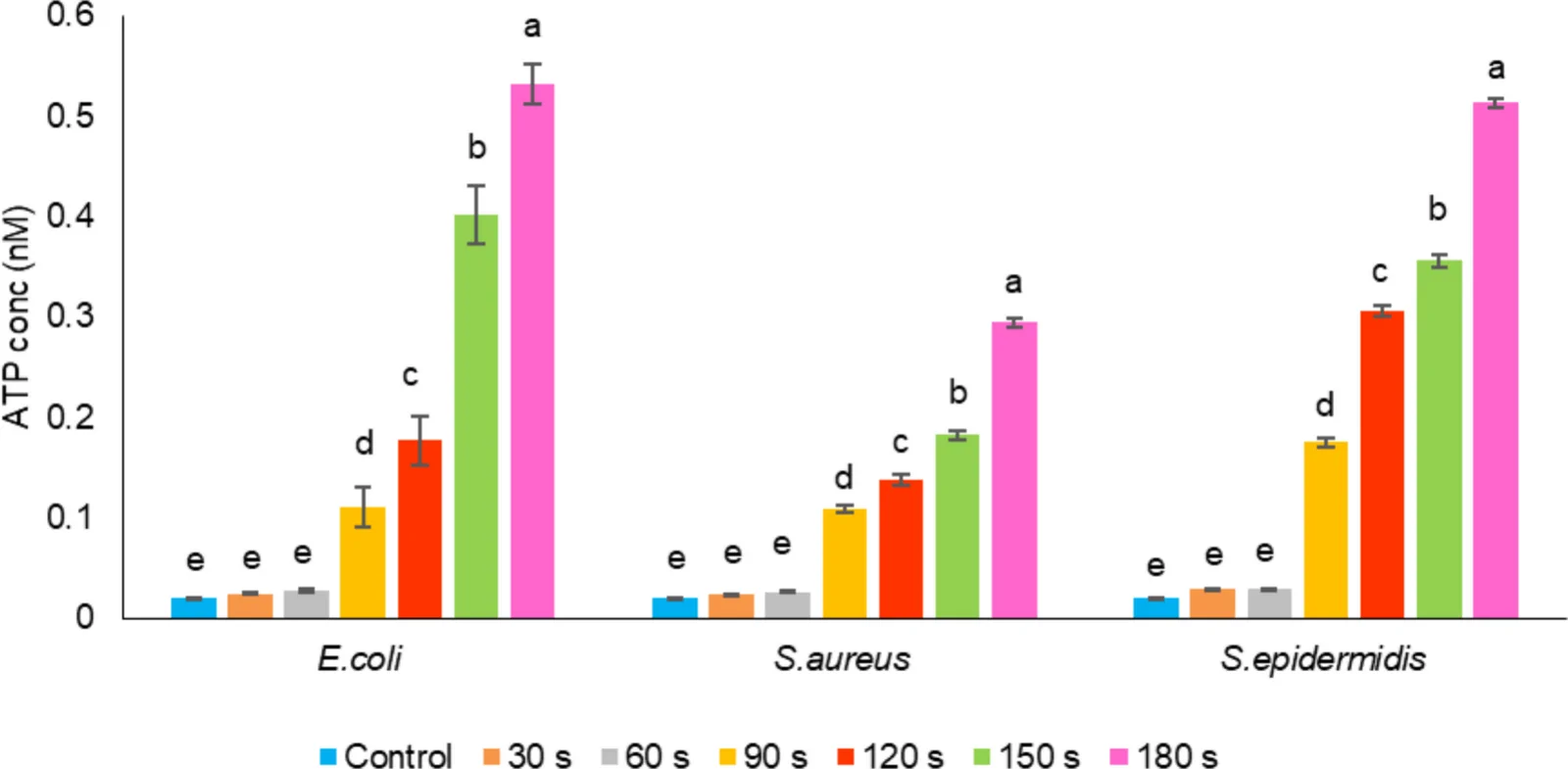
ATP in cell culture supernatant post CAP treatment in *E*. *coli* P4, *S. aureus* M60, and *S. epidermidis* NCTC11047. CAP was applied for a duration of 30–180 s at a distance of 1 cm. CAP-untreated samples served as the controls. The bars in the graph represent the mean concentration of ATP with standard deviation indicated by error bars, derived from three independent experiments, each conducted in triplicate (*n* = 9). Bars with different superscript letters differ significantly (*p* < 0.05) within each bacterial group

The effect of CAP on bacterial cell integrity was further investigated by quantifying the amount of extracellular ATP in the bacterial supernatant after CAP treatment. CAP treatment for 30–60 s did not show any significant change in the ATP levels in bacterial suspensions. However, a surge in ATP levels was noted after 90 s. ATP levels were significantly higher from 90 to 180 s in all bacterial strains as compared to the control, with the highest ATP concentration observed after 180-s CAP treatment, indicating a treatment time-dependent increase (Fig. 7). Although all the bacterial strains showed a similar trend, the ATP levels in *S. aureus* were significantly lower (*p* < 0.05) than *S. epidermidis* and *E. coli*.

### Effect of CAP pretreatment on antibiotic efficacy

To evaluate whether CAP pretreatment enhanced the antibiotic susceptibility, *E. coli*, *S. aureus*, and *S. epidermidis* were pretreated with CAP for 30 s. As shown in the Antimicrobial efficacy of CAP on a collagen-elastin matrix section, this exposure duration did not produce a significant reduction in bacterial log CFU/cm^2^ and therefore was classified as sub-lethal. Following CAP treatment, antibiotic susceptibility assay with engemycin and enrofloxacin revealed notable shifts in MIC and MBC values in *E. coli*, *S. aureus*, and *S. epidermidis* when compared with untreated controls (Table 1). In *E. coli*, *S. aureus*, and *S. epidermidis*, CAP pretreatment resulted in a 50% reduction in MIC and a 75% reduction in MBC values for engemycin. In the case of enrofloxacin, *E. coli* exhibited a 50% reduction in both MIC and MBC, while *S. aureus* and *S. epidermidis* showed a 50% reduction in MIC and a 75% reduction in MBC. These findings indicate CAP pretreatment may enhance the efficacy of antibiotics, demonstrating a higher antimicrobial effect when antibiotics are combined with CAP.

**Table 1:** Minimum inhibitory concentration (MIC) and minimum bactericidal concentration (MBC) values of engemycin and enrofloxacin for untreated bacteria and bacteria treated at sub-lethal CAP treatment time (*n* = 9)

Bacteria	Treatment	Engemycin (µg/mL)		Enrofloxacin (µg/mL)	
		MIC	MBC	MIC	MBC
*E. coli* P4	Control	2	32	0.25	0.5
	30-s CAP	1	8	0.125	0.25
*S. aureus* M60	Control	1	8	0.5	1
	30-s CAP	0.25	1	0.25	0.25
*S. epidermidis* NCTC11047	Control	125	250	0.5	1
	30-s CAP	62.5	125	0.25	0.25

## Discussion

CAP generated via piezoelectric direct discharge represents a novel CAP technology that operates at low temperatures under energy-efficient atmospheric air-based conditions (Konchekov et al. 2024). Its potential application extends to veterinary medicine (Yoo et al. 2023), where wound infections remain a significant challenge in livestock management. Therefore, in this study, the antimicrobial activity of CAP was evaluated against Gram-positive and Gram-negative bacteria relevant to the bovine wound environment, including *E. coli* P4 and *S. aureus* M60 isolated from dairy cattle and *S. epidermidis* NCTC 11047 as an opportunistic skin-associated pathogen to assess its potential for decontaminating cattle wounds. A collagen-elastin matrix membrane inoculated with bacteria served as a skin-mimicking model, enabling assessment under laboratory conditions that resemble dermal structure. Several studies have demonstrated the antimicrobial efficacy of CAP. For example, Rasouli et al. (2022) reported that pulse spark discharge and arch discharge (air as gas feed) application for 2–8 min resulted in a significant reduction by 6 and 4.9 log CFU/mL in *Pseudomonas aeruginosa* and *S. aureus* as compared to the initial 7 log CFU/mL in the untreated control. Similarly, Nima et al. (2021) observed an exponential reduction in *S. mutans* biofilms following CAP jet (argon as working gas) exposure for 30–150 s, with the highest antimicrobial effect observed at 120 s. Schaal et al. (2025) further demonstrated a 3–4.5 log CFU/mL reduction in *S*. *aureus*, *S. epidermidis*, *E. coli*, *P. aeruginosa*, and *Candida albicans* following CAP-Aerosol (distilled water activated by plasma-enriched air) treatment using air as the working gas.

The present study determined the optimal parameters for CAP application in terms of treatment time and treatment distance. The data obtained in this study clearly demonstrate that CAP generated using piezoelectric direct discharge technology exhibits strong antibacterial efficacy against the tested bacteria, with inactivation increasing as a function of exposure duration. Consistent with this, Lim et al. (2025) reported a 3.8 log reduction in *S. mutans* biofilms cultured in microtiter plates following 15 min of corona discharge CAP (air as gas feed), with a 1-min treatment still producing a 1.79 log reduction. Similarly, Patel et al. (2025) observed a complete 7 log CFU/mL reduction of *E. coli* on agar plates following 10 min of dielectric-barrier discharge (DBD) CAP treatment. Han et al. (2016) reported that 3 min of DBD-CAP exposure (atmospheric air as gas feed) reduced *E. coli* and *S. aureus* counts to 2.5 and 2.4 log CFU/mL, respectively, with 5 min of treatment reducing both below the detectable limit. Studies employing a collagen-elastin matrix further support these findings. Boekema et al. (2021) reported a 4 log reduction of methicillin-resistant* Staphylococcus aureus* (MRSA) on CEM following 2-min DBD-CAP treatment. Likewise, Dijksteel et al. (2020) demonstrated complete inactivation of *P. aeruginosa* on CEM inoculated with 7 log CFU/mL following 2 min of surface DBD-CAP exposure (air as gas feed), consistent with the outcomes observed in the current study.

In the present study, the bactericidal efficacy increased with CAP exposure at both tested distances (1 and 2 cm), confirming antimicrobial activity under both exposure conditions. However, the overall effect was attenuated at 2 cm. No antimicrobial activity was observed at this distance up to 60 s of CAP exposure. Significant reductions in bacterial load were evident only after 90 s of treatment for *E. coli* and *S. epidermidis*, whereas *S. aureus* required longer exposure durations (120–180 s) to achieve a statistically significant antimicrobial effect. Based on these findings, a treatment distance of 1 cm was determined to be optimal for effective bacterial elimination. This distance-dependent decrease in antimicrobial activity is consistent with findings reported by Dahle et al. (2024). Their study demonstrated that increasing the treatment distance between a gliding arc plasma jet CAP source and *E. coli* and *S. aureus* biofilms grown on glass coupons resulted in a reduction in antimicrobial efficacy. Specifically, when the distance was increased from 1 to 10 mm, bacterial reduction decreased from 6.4 to 3.0 log CFU/cm^2^ in *E. coli* and from 3.6 to 2.8 log CFU/cm^2^ in *S. aureus*. Similar distance-dependent effects were reported by Spiegel et al. (2024), who observed bacterial counts below the detection limit at a distance of 1 cm, while survival increased significantly to 5.4 log CFU when the distance was increased to 2 cm. Pedroni et al. (2018) likewise reported reduced antimicrobial efficacy with increasing treatment distance using an atmospheric pressure plasma jet (air as gas source), with *E. coli* reduction to 2.5 log CFU at 0.5 cm compared to 1.2 log CFU at 2 cm. The reduced antimicrobial activity at 2 cm can be attributed to the dilution of the reactive oxygen and nitrogen species (RONS) in the surrounding atmosphere before they reach the bacterial surface (Das et al. 2022). As the plasma-target distance increases, reactive species are more likely to interact with atmospheric chemical species, lowering their effective concentration at the target site. This mechanism is supported by several studies. Taheri et al. (2024) reported a reduction in RONS concentration when the treatment distance between aqueous media and DBD CAP (argon gas source) was increased from 7 to 21 mm, while Lotfy et al. (2020) documented a decrease in the emission intensity of multiple reactive species (OH, OI, N_2_^+^) as the distance from the plasma source increased from 7 to 13 mm. Similarly, Mashayekh et al. (2015) demonstrated reduced concentration of OH, O_3_, and O_2_ radicals when the treatment distance increased from 1 to 2 cm, attributing this effect to reactive species interacting with atmospheric gases. Importantly, Dahle et al. (2024) further showed that increasing the distance between the plasma and the target (bacteria) alters the reactive species composition, with short-lived, highly reactive OH radicals dominating at shorter distances (1–5 mm) and long-lived species such as NO and H becoming more prevalent at greater distances (5–10 mm). Collectively, these distance-dependent changes in both RONS concentration and composition provide mechanism-based evidence for the reduced antimicrobial activity observed at longer plasma-target distances. Contrasting observations have been reported by Abonti et al. (2016), who observed higher antimicrobial activity at a longer distance (20 mm) compared to 2 mm during multi-gas CAP jet treatment of *S. mutans*, *Limosilactobacillus fermentum*, and *Aggregatibacter actinomycetemcomitans*. They proposed that increased plasma-air interaction at longer distances promoted the formation of additional reactive species capable of bacterial inactivation. Nevertheless, the majority of available evidence, including the present study, supports the conclusion that shorter plasma-target distance generally produces greater antimicrobial efficacy. In the current study, CAP treatment of 180 s at 1 cm distance reduced the bacterial count of *E. coli*, *S. aureus*, and *S. epidermidis* below the detection limit (1 log CFU/cm^2^). In contrast, at a distance of 2 cm, although *E. coli* and *S. epidermidis* counts fell below the detection limit, *S. aureus* remained detectable, indicating greater tolerance to CAP at a longer distance compared with the other species. The difference in susceptibility of *S. aureus* and *E. coli* to CAP treatment can be attributed to their distinct cell wall architectures (Mahmoud et al. 2025). *S. aureus*, a Gram-positive bacterium, possesses a thick, multilayered peptidoglycan structure that not only provides mechanical strength but also serves as an effective barrier against oxidative damage from reactive RONS, thereby limiting CAP-induced injury (Mai-Prochnow et al. 2016). In contrast, *E. coli*, a Gram-negative bacterium, has a thinner peptidoglycan layer located between the inner cytoplasmic membrane and an outer membrane containing lipopolysaccharide. This outer membrane is more permeable to small molecules and RONS, and the reduced peptidoglycan thickness offers less structural protection, rendering *E. coli* more susceptible to the RONS generated during CAP exposure (Mohseni et al. 2023).

Previous studies have reported that bacterial cells can enter a viable but non-culturable (VBNC) state in response to CAP exposure (Liao et al. 2021; Zhang et al. 2023a). In this state, bacteria exhibit reduced metabolic activity and altered intracellular energy allocation, leading to delayed or impaired growth on culture media, with potential underestimation of viability in CFU-based assays. To strengthen the findings from CFU enumeration (Antimicrobial efficacy of CAP on a collagen-elastin matrix section) and to exclude the possibility that the reduced CFU counts were due to VBNC induction, CAP efficacy was additionally evaluated using a live/dead fluorescence staining assay. This complementary method not only corroborated the antimicrobial effect observed in CFU analysis but also offered preliminary evidence of CAP-induced membrane damage, thereby guiding the design for subsequent mechanistic investigations. Data from the present study demonstrate that the reduction in bacterial viability was dose-dependent and increased with increased treatment time. These findings align with Xu et al. (2018), who reported a significant reduction in *E. coli* and *S. aureus* viability after 20 min of direct current air CAP treatment. Similarly, Patange et al. (2021) observed increased PI fluorescence with increased DBD CAP exposure time, indicating a significant proportion of dead cells within biofilms. Consistent with present findings, exposure to DBD CAP (helium and oxygen as working gases) has been reported to reduce *Lentilactobacillus hilgardii* viability by 8.1% after 10 min and 98.8% after 15 min, highlighting the effect of prolonged treatment duration on bacterial inactivation (Niedźwiedź et al. 2020). However, it should be noted that the viability reduction reported by Niedźwiedź et al. 2020 was derived from a fluorescence staining method that characterises bacterial physiological states, rather than the live/dead staining employed in the current study. The observed increase in PI fluorescence, indicative of cell death, is attributable to CAP-induced damage to bacterial cell membranes and cell wall mediated by plasma-generated reactive species, including oxygen radicals, hydroxyl, and hydrogen peroxide, which impart oxidative stress on bacterial cells (Khalaf et al. 2024). These RONS can erode the cell wall by cleaving C-O glycosidic bonds in peptidoglycan (Mai-Prochnow et al. 2016; Borges et al. 2021) and induce pore formation in the cell membrane (Lunder et al. 2024), thereby rendering injured cells more susceptible to oxidative attack. In the present study, CAP treatment progressively reduced viability in all bacterial strains, with *E. coli* exhibiting the most pronounced decrease, while *S. aureus* retained higher levels of viable cells than *E. coli* and *S. epidermidis*, consistent with the CFU-based findings (Fig. 1).

In the present study, a piezoelectric direct discharge plasma generator was employed. Previous studies utilising the same technology have demonstrated the production of reactive species such as ozone (O_3_), nitrogen dioxide (NO_2_), nitric oxide (NO), and hydrogen peroxide (H_2_O_2_) (Korzec et al. 2021; Oliinychenko et al. 2025). These species are known to exert antimicrobial effects through distinct mechanisms. H_2_O_2_ oxidises DNA, proteins, and membrane lipids, causing oxidative damage (Abdelshafy et al. 2024), while O_3_ damages the bacterial cell membrane (Banerjee et al. 2024). NO inactivates the bacterial membrane protein, targeting the iron-sulphur centres in the enzymes, and targets the respiratory system of bacteria, disrupting the proton motive force essential for bacterial energy generation. In addition, nitrogen dioxide exerts antimicrobial activity by initiating peroxidation of the membrane, protein nitration, and DNA damage (Roberts et al. 2024).

The concentration of intracellular ROS generated is known to be influenced by multiple parameters, such as the type of gas used, the distance between the plasma source and the target, applied voltage, frequency, and treatment duration (Cheng et al. 2020). In the current study, CAP exposure for 30–60 s induced high intracellular ROS levels in all bacteria, followed by a decline as the treatment time increased. These findings are in agreement with Lv and Cheng (2022), who reported elevated ROS levels in *S. typhimurium* suspension following 1–3 min DBD CAP (air as gas feed), with subsequent decreases at longer exposure times. Similarly, Pan et al. (2022) observed a reduction in fluorescence intensity from 52.9 to 42.1% in 7 log CFU/mL of *Listeria monocytogenes* suspension when the DBD CAP treatment time was increased from 6 to 8 min. Irrespective of the type of plasma used in these studies, all exhibited the same pattern of elevated ROS followed by a decline. The mechanism underlying this decline has not been definitively established; however, several hypotheses may be considered. One plausible explanation is that prolonged CAP exposure leads to bacterial cell lysis, resulting in the dissipation or leakage of intracellular ROS into the surrounding medium, thereby reducing the detectable intracellular signal (Lv & Cheng 2022). An alternative hypothesis relates to the ROS detection methodology itself, which is that extended CAP treatment may cause denaturation and inactivation of intracellular enzymes, including esterase, which catalyses the conversion of 2′,7′-dichlorodihydrofluorescein diacetate (H_2_DCFDA) to 2′,7′-dichlorodihydrofluorescein (H_2_DCF), and its further oxidation, thereby facilitating its retention in the bacterial cell (Lv & Cheng 2022).

Bacterial membrane lipids are highly vulnerable to the RONS generated during CAP exposure. Lipid oxidation produces reactive aldehydes, most notably MDA, which acts as a secondary messenger, further propagating the oxidative damage to proteins and other macromolecules in bacterial cells (Zhang et al. 2023a). Therefore, to assess CAP-induced oxidative damage to bacterial lipids following CAP treatment, the present study quantified MDA, a marker of lipid peroxidation. Across all strains, CAP exposure resulted in significantly elevated MDA concentrations compared with untreated controls, with levels increasing in a treatment time-dependent manner. These findings are in alignment with Colagar et al. (2017), who reported a progressive increase in MDA concentration in *E. coli* suspensions that were subject to APPJ CAP (argon and air as gas feed) over an exposure time of 2.5 to 12.5 min, with the highest MDA concentration observed at 12.5 min. Consistent trends have been reported in other studies, where MDA concentration in *S. typhimurium* increased with prolonged air DBD-CAP treatment, reaching a maximum of more than 0.6 nmole/mL after 5-min treatment (Lv and Cheng 2022). Extended argon plasma jet treatment (10–40 min) significantly elevated MDA levels in *E. coli* suspensions, with peak concentration exceeding 45 nM after 40-min exposure (Dolezalova and Lukes 2015). Similarly, short-duration DBD-CAP exposure (air as gas feed; 0–60 s) has been shown to increase MDA levels in *E. coli* by 200% relative to untreated controls (Joshi et al. 2011)*.* The time-dependent rise in MDA is in line with increased RONS flux at longer exposures (Zhang et al., 2023a). The highest MDA response in the present study was observed in *E. coli* relative to *S. aureus* and *S. epidermidis*, consistent with the findings of Olatunde et al. (2019), who reported higher MDA levels in *E. coli*, *P. aeruginosa*, and *Vibrio parahaemolyticus* as compared to *L. monocytogenes* and *S. aureus* following 5 min of DBD-CAP treatment. This particular trend is attributable to the higher lipid content and outer-membrane susceptibility characteristic of Gram-negative bacteria. Together, the data support lipid peroxidation as one of the mechanisms underlying CAP-mediated membrane damage, with the magnitude of injury dependent on exposure duration.

CAP-induced cellular damage compromises bacterial membrane integrity, causing leakage of cytoplasmic biomolecules essential for metabolic functions of the bacterial cell (Nwabor et al. 2022; Mohseni et al. 2023). Among these, ATP and LDH are key intracellular components essential for bacterial metabolism and cellular homeostasis, and they play a crucial role in energy generation. In the present study, both ATP and LDH were detected in the extracellular supernatant of the bacterial culture following CAP treatment, providing direct evidence of CAP-induced membrane permeabilisation. The concentrations of extracellular ATP and LDH in CAP-treated samples were significantly higher than those in untreated controls and increased progressively with treatment duration, indicating a time-dependent loss of bacterial structural integrity. The highest ATP and LDH levels were observed following 180 s of CAP exposure, reflecting maximal membrane disruption at prolonged treatment time. These findings are consistent with Maybin et al. (2023), who reported extracellular ATP and LDH levels in *P. aeruginosa* PAO1 suspensions following DBD-CAP (air, 0–120 s) treatment, with concentrations increasing with exposure time and peaking at 120 s. Further supporting evidence for this mechanism is provided by Kvam et al. (2012), who demonstrated significant depletion of intracellular ATP in MRSA, *P. aeruginosa*, and *C. albicans* following 1 s DBD-CAP (argon as gas source), with further reduction observed at 7 and 30 s relative to the untreated control. This progressive intracellular ATP loss is indicative of CAP-induced membrane permeabilisation and cellular damage, facilitating ATP efflux. In support of this mechanism, multiple studies have also reported CAP-induced leakage of intracellular macromolecules, including proteins and DNA, providing further evidence of compromised bacterial integrity (Lu et al. 2014; Liew et al. 2023; Hu et al. 2025). Related evidence has been reported using plasma-activated water (PAW), for instance, Sun et al. (2024) demonstrated a 38–65% reduction in intracellular ATP in five strains of *Salmonella Newport* following PAW treatment. Although PAW was used instead of direct CAP exposure, the observed intracellular ATP depletion, indicating ATP leakage, is consistent with the present finding, further supporting membrane permeabilisation as one of the features of CAP-mediated bacterial inactivation.

Importantly, this study evaluated whether brief exposure to CAP potentiates antibiotic activity. Two broad-spectrum agents were tested: (i) engemycin (oxytetracycline), widely used in veterinary settings for the treatment of wound infections in cattle, including those associated with the dehorning procedure (Hund et al. 2020), and (ii) enrofloxacin, a fluoroquinolone indicated for respiratory and systemic infections (Grabowski et al. 2022), representing a different antibiotic class with a distinct mechanism of action as compared to engemycin. Inclusion of these two antibiotics allowed evaluation of CAP-induced changes in bacterial susceptibility across antibiotics with different modes of action. Bacterial cultures were pretreated with CAP for 30 s (a dose that did not produce any significant reduction in bacterial count in viability screens) and subsequently challenged with each antibiotic. CAP pretreatment produced a ≥2-fold reduction in both MIC and MBC for oxytetracycline and enrofloxacin relative to untreated controls, indicating that substantially lower antibiotic doses were required to inhibit growth and achieve bactericidal endpoints in CAP-conditioned cells. These findings are concordant with previous reports of CAP antibiotic synergy studies. Maybin et al. (2023) demonstrated reduced MIC/MBC values for tobramycin, gentamicin, ciprofloxacin, and chlorhexidine in *P. aeruginosa* PAO1 after a 45-s CAP exposure in planktonic cells and 90 s for biofilms, attributing this effect to CAP-induced morphological and envelope changes supported by transcriptomic up-regulation of cell-structure proteins (e.g. MreB, LptF, OprG). Unlike Maybin et al. (2023), who employed CAP as a synergistic therapeutic approach in combination with antibiotics to enhance the eradication of planktonic cells and biofilm, the present study applied CAP as a pretreatment to investigate its impact on subsequent antibiotic susceptibility. Specifically, bacterial cells were exposed to a 30-s CAP treatment that did not produce a significant reduction in CFU/cm^2^, indicating cellular perturbation rather than direct killing. This experimental design assumes that CAP pretreatment induced physiological and structural stress responses sufficient to alter cellular susceptibility without causing cell death. Related observations have been reported in other studies. Kvam et al. (2012) documented increased surface permeability and membrane damage in planktonic MRSA, *Candida albicans*, and *P. aeruginosa* following CAP treatment. Guo et al. (2018) reported decreased MIC/MBC values across seven antibiotic classes in MRSA after 30–40-s sub-lethal DBD-CAP (helium, nitrogen, and oxygen gas mixture) treatment, with greater reductions at longer exposure and elimination of CAP-treated persisters in the presence of antibiotics. At the ultrastructural level, short CAP exposures can rapidly remodel bacterial envelopes. This interpretation is further supported by the findings of Eced-Rodriguez et al. (2024), who demonstrated that exposure of *B. cereus* to moderate-intensity DBD-CAP (100–200 W for 5 min) induced surface poration, erosion, and excavation mediated by reactive species, without causing bacterial lysis. These conditions were classified as sub-lethal, as the treatment did not inactivate the bacterial cells, and bacterial viability was shown to recover during subsequent storage. In contrast, higher-intensity treatment (300 W for 5 min) resulted in severe structural damage, including cell flattening, collapse, and leakage of intracellular contents, consistent with lethal membrane disruption. These observations indicate that low-intensity or sub-lethal CAP exposure induces structural and physiological variations that are sufficient to alter bacterial function without causing immediate inactivation, including changes in gene expression, membrane permeability, and metabolic regulations (Sun et al. 2024), thereby rendering bacteria more physiologically vulnerable and responsive to subsequent antibiotic exposure. Consistent with this framework, Patinglag et al. 2021), reported heterogeneous populations with membrane defects in *E. coli* and *P. aeruginosa* after 3–5-s CAP treatment, while Huang et al. (2020) reported surface shrinkage, wrinkling, and indentation in *Salmonella typhimurium* at 50 s and extensive loss of integrity at 100 s of CAP exposure. Beyond structural damage, low-dose CAP also imposes oxidative stress (Yang et al. 2021b), with downstream repercussions on transport proteins (porins/efflux pumps), quorum sensing, virulence factor expression, and membrane functionality (Paldrychová et al. 2020). Taken together, our data support that sub-lethal CAP exposure enhances bacterial susceptibility to two antibiotic classes, likely via combined envelope permeabilisation, transport dysregulation, and stress-response disruption that increase intracellular antibiotic accumulation and/or retention.

## Conclusion

This study demonstrates that CAP generated by a piezoelectric technology, utilising atmospheric air, exerts broad antimicrobial activity against Gram-positive and Gram-negative bacteria isolated from dairy cattle. Using a bovine collagen-elastin dermal matrix to approximate wound tissue, we identified exposure conditions that reduced viable counts to below the detection limit across *E. coli* P4, *S. aureus* M60, and *S. epidermidis* NCTC 11047. The mechanistic readouts, including increased intracellular ROS and leakage markers (ATP, LDH), are consistent with oxidative injury and loss of membrane integrity. Importantly, brief sub-lethal CAP pre-exposure significantly lowered the MIC/MBC of oxytetracycline and enrofloxacin, supporting a CAP-antibiotic sequence as a dose-sparing strategy. Such potentiation aligns with antimicrobial-stewardship goals, supporting the prospect of reduced antibiotic inputs while maintaining efficacy in future validated applications. Despite these findings, the present study is based on in vitro experiments, and the bacterial strains investigated may not fully represent the diversity of wound-associated pathogens encountered in bovine settings. Nevertheless, CAP represents a practical, portable and potentially sustainable adjunct for on-farm wound hygiene and therefore future work should confirm safety and effectiveness in vivo under field conditions. Subsequently, studies aimed at elucidating strain/biofilm variability, and the molecular basis of antibiotic sensitisation, including effects on bacterial membrane permeability and transport system, will further advance understanding of the antimicrobial mechanisms of CAP. In parallel, compatibility studies with additional drug classes, tissue-tolerability assays, and assessment of treatment logistics will be essential to support translation to veterinary practice.

## Data Availability

The data that support the findings of this study are not openly available but are available from the corresponding author upon reasonable request.
